# Data in support of toxicity studies of structurally modified plant virus to safety assessment

**DOI:** 10.1016/j.dib.2018.10.102

**Published:** 2018-10-26

**Authors:** N.A. Nikitin, V.A. Zenin, E.A. Trifonova, E.M. Ryabchevskaya, M.S. Yurkova, O.A. Kondakova, A.N. Fedorov, J.G. Atabekov, O.V. Karpova

**Affiliations:** a Department of Virology, Faculty of Biology, Lomonosov Moscow State University, 1-12 Leninskie Gory, Moscow 119234, Russian Federation; b Group of Molecular Biotechnology, Federal State Institution “Federal Research Centre “Fundamentals of Biotechnology” of the Russian Academy of Sciences”, 33-2 Leninsky pr., Moscow 119071, Russian Federation

**Keywords:** Plant virus, Tobacco mosaic virus, Spherical particles, Toxicity

## Abstract

This data article is related to the research article entitled “Assessment of structurally modified plant virus as a novel adjuvant in toxicity studies” (Nikitin et al., 2018), devoted to the safety study of structurally modified plant virus - spherical particles (SPs). SPs are generated by thermally denatured tobacco mosaic virus (TMV) coat protein and act as effective adjuvant for development of new vaccine candidates. This article reports the additional results on the toxicity studies of TMV SPs. The weight coefficients of laboratory animals internal organs complements the data of the subchronic toxicity studies. Also plaque-forming cell assay, delayed-type hypersensitivity test and peritoneal macrophage assay as a part of immunotoxicity studies of TMV SPs are presented.

**Specifications table**

**Table:** 

Subject area	*Biology*
More specific subject area	*Toxicology, Plant virus*
Type of data	*Table*
How data was acquired	*Analytical balance HR-250AZ (AND, Japan). Phagocytic index was counted with a Vybrant Phagocytosis Assay Kit (Thermo Fisher Scientific, USA) on BX43 system microscope (Olympus, Japan).*
Data format	*Analyzed*
Experimental factors	*Spherical particles production by thermal transition of TMV, intramuscular injections of TMV SPs in experimental animals*
Experimental features	*Registration of laboratory animals response on TMV SPs administration*
Data source location	*Department of Virology, Lomonosov Moscow State University,1–12 Leninskie gory, Moscow 119234, Russia*
Data accessibility	*Data are provided with this article*
Related research article	*Nikitin N.A., Zenin V.A., Trifonova E.A., Ryabchebskaya E.M., Kondakova O.A., Fedorov A.N., Atabekov J.G., Karpova O.V. (2018) Assessment of structurally modified plant virus as a novel adjuvant in toxicity studies. Regulatory Toxicology and Pharmacology. Vol. 97, 127–133*

**Value of the data**

• These data are useful to demonstrate that TMV SPs, a potential vaccine adjuvant, is not toxic in laboratory animals.
• The data demonstrate the absence of immunotoxicity or deep overload of immune functions in mice as well as alterations in weight after TMV SPs administration.
• The data are useful for further estimation of novel adjuvant safety.
• These data are valuable to researchers interested in plant viruses and their derivatives for biotechnological and medical application.

## 1 Data

An essential step for the novel universal adjuvant development is to study their safety on laboratory animals. In this article we display additional data on the weight coefficients of the internal organs within the subchronic toxicity studies and immunotoxicity studies of spherical particles based on structurally modified helical plant virus, including plaque-forming cell assay, delayed-type hypersensitivity test and peritoneal macrophage assay.

## 2 Experimental design, materials, and methods

### 2.1 Animals

Young adult laboratory animals were used: Wistar outbred rats (140–160 g, 7–8 weeks old), Standard Chinchilla rabbits (3.2–3.5 kg, 5–6 months old), F1 (CBA x C57BL/6) hybrid mice (20–22 g, 8–9 weeks). Animal studies were performed under protocols approved by the Federal Research Centre of Biotechnology of the Russian Academy of Sciences Animal Ethics Committee (Ethics Committee Session No. 170511), in accordance with national law and Directive 2010/63/EU of the European Parliament and of the Council of 22 September 2010 on the protection of animals used for scientific purposes.

### 2.2 Structural modification of plant virus (TMV)

TMV SPs were obtained according to Refs. [2], [3]. TMV SPs size was characterized by transmission electron microscopy and nanoparticle tracking analysis, as described previously [4], [5]. For all experiments, TMV SPs were buffered with apyrogenic PBS sterile solution at concentration of 1 mg/ml.

### 2.3 Study design

#### 2.3.1 Analysis of internal organs weight

To evaluate the weight coefficients of the internal organs following three consecutive intramuscular injections, at two-week intervals, TMV SPs in low (20 µg per animal) and high (200 µg per animal) doses were administered on rats and rabbits as described in Ref. [1]. Phosphate buffer saline was used as a control. Each group of rodents contained five males and five females, while each group of rabbits consisted of three males and three females. The groups were numbered sequentially. Animals were euthanized on the 42nd day, and an autopsy was undertaken. Organs were weighed to the nearest mg with analytical balance HR-250AZ (AND, Japan) and organ/body weight ratio was calculated (Table 1).

### 2.3.2 Immunotoxicity

Immunotoxicity evaluation was performed in compliance with federal regulatory requirements. F1 hybrid mice (CBA x C57BL/6) in three groups (10 or 100 μg of SPs in PBS IM or 100 μl PBS IM as a control), consisting of five males and five females in each group, were used. There were three tests, thus 90 mice (20–22 g) were used. Humoral-mediated immunity was assessed through T-dependent antibody response. The plaque-forming cell (PFC) antibody response to sheep red blood cells (SRBC), or the plaque assay, was chosen to analyze the effects on T-dependent antigen response. Mice were administered with doses of SPs or PBS, as indicated above, one hour before SRBC immunization, and were euthanized five days after. Then, a standard PFC protocol was performed [6]. PFCs per spleen were calculated.

Delayed-type hypersensitivity (DTH) as an *in vivo* assay of cell-mediated immune function was used [7]. DTH reaction was induced by trinitrobenzenesulfonate (TNBS) treatment [8]. The study and control groups were treated with SPs and PBS respectively an hour after TNBS immunization (200 μl 10 μM sterile TNBS solution in isotonic sodium chloride subcutaneously). Six days later, the right forepaw footpads were injected with 50 μl of 10 μM sterile TNBS solution in isotonic sodium chloride, and the left footpads with isotonic sodium chloride solution alone. Footpad swelling was estimated by footpad diameter measurement [9], and using cross-sectional area calculation. Footpad cross-sectional area was approximated by ellips area formula. The semi-major axis was half of the maximal width (measured with vernier caliper (ChIZ, Russia) to the nearest 0,02 mm) and the semi-minor axis was the half of perpendicular measurement. The swelling index was calculated as the right/left footpad cross-sectional area ratio, as a percentage (Table 2).

**Table 1:** The weight coefficients of the internal organs in repeated dose toxicity study. Groups 1, 4 are controls (PBS), groups 2, 5 - low dose of TMV SPs (20 µg) and groups 3, 6 - high dose of TMV SPs (200 µg). Rats (groups 1, 2, 3); rabbits (groups 4, 5, 6). Mean value and SD were calculated with IBM SPSS Statistics (23.0.0.0 64 bit for Windows); no significant difference by one-way ANOVA was detected.

	**Sex**	**Group 1**	**Group 2**	**Group 3**	**Group 4**	**Group 5**	**Group 6**
**Lungs**	Female	0.531 ± 0.0703	0.461 ± 0.0409	0.467 ± 0.0857	0.481 ± 0.1011	0.463 ± 0.1365	0.457 ± 0.0654
	Male	0.513 ± 0.0596	0.527 ± 0.0460	0.527 ± 0.0438	0.451 ± 0.0683	0.471 ± 0.0375	0.475 ± 0.1273
**Heart**	Female	0.361 ± 0.0563	0.337 ± 0.0620	0.356 ± 0.0532	0.174 ± 0.0032	0.164 ± 0.0067	0.172 ± 0.0035
	Male	0.329 ± 0.0580	0.343 ± 0.0542	0.289 ± 0.1045	0.171 ± 0.0067	0.172 ± 0.0174	0.170 ± 0.0165
**Liver**	Female	2.982 ± 0.2740	2.756 ± 0.4644	3.140 ± 0.4887	4.228 ± 0.2940	3.645 ± 0.4717	3.320 ± 0.4688
	Male	2.849 ± 0.3916	2.808 ± 0.3248	2.524 ± 0.3103	3.841 ± 0.2074	4.020 ± 0.5148	3.945 ± 0.4476
**Thymus**	Female	0.126 ± 0.0115	0.150 ± 0.0076	0.123 ± 0.0159	0.083 ± 0.0128	0.095 ± 0.0081	0.087 ± 0.0061
	Male	0.132 ± 0.0106	0.120 ± 0.0173	0.129 ± 0.0123	0.078 ± 0.0089	0.088 ± 0.0131	0.090 ± 0.0104
**Kidneys**	Female	0.703 ± 0.0383	0.708 ± 0.0251	0.719 ± 0.0439	0.439 ± 0.0154	0.449 ± 0.0187	0.442 ± 0.0065
	Male	0.701 ± 0.0543	0.730 ± 0.0331	0.709 ± 0.0288	0.417 ± 0.0289	0.429 ± 0.0176	0.433 ± 0.0153
**Spleen**	Female	0.145 ± 0.0944	0.238 ± 0.0656	0.214 ± 0.0557	0.199 ± 0.0093	0.191 ± 0.0178	0.190 ± 0.0078
	Male	0.222 ± 0.0397	0.210 ± 0.0328	0.187 ± 0.0685	0.198 ± 0.0097	0.181 ± 0.0115	0.203 ± 0.0191

**Table 2:** Immunotoxicity study results. Arithmetical mean are presented. The plaque-forming cell (PFC) is an index of T-dependent antibody response, the swelling index is a function of cell-mediated immune response and the phagocyte concentration and phagocytic index represents macrophage activity. F1 hybrid mice (CBA x C57BL/6) in three groups (10 and 100 μg of SPs in PBS intramuscular (IM) or 100 μl PBS IM) were used. Mean value and SD were calculated with IBM SPSS Statistics (23.0.0.0 64 bit for Windows); no significant difference by one-way ANOVA was detected.

	**PFC**	**Swelling index**	**Phagocyte conc. 10**^**3**^**at ml**	**Phagocytic index**
**Control**	140,457 ± 4924	97.4 ± 10.6	1526 ± 120.8	3.18 ± 0.430
**Low dose**	142,159 ± 5192	99.7 ± 12.6	1540 ± 101.3	2.74 ± 0.390
**High dose**	142,490 ± 4352	85.0 ± 29.6	1599 ± 152.1	2.84 ± 0.593

The peritoneal macrophage assay was used for phagocytic activity testing. Murine immune cells were isolated using a common protocol [10], and then their activity was studied with a Vybrant Phagocytosis Assay Kit (Thermo Fisher Scientific, USA). Five replicates on each animal were used. The number of harvested cells and phagocytosis assay result were measured.
